# Horseshoe Kidney With a Documented Giant Calculi: A Case Report

**DOI:** 10.7759/cureus.29144

**Published:** 2022-09-14

**Authors:** Geetika Malhotra, Abhijit Dhale, Jay D Dharamshi

**Affiliations:** 1 Department of General Surgery, Jawaharlal Nehru Medical College, Datta Meghe Institute of Medical Sciences, Wardha, IND; 2 Department of Urology, Jawaharlal Nehru Medical College, Datta Meghe Institute of Medical Sciences, Wardha, IND

**Keywords:** renal calculi, urolithiasis, d.j stenting, pyelolithotomy, horseshoe kidney

## Abstract

The horseshoe kidney is the most frequent genitourinary fusion abnormality. The horseshoe kidney is a combination of the anatomical abnormalities of ectopia and malrotation. Along with other anomalies, it is linked to malrotations, fluctuating blood flow, high ureter insertion, a tendency to establish a ureteropelvic junction, and blockage in up to one-third of patients, and these are all symptoms of this condition. Kidney calculus and pelvic ureteric junction (PUJ) obstruction are one of horseshoe kidneys' most prevalent side effects and are seen in approximately one-third of the patients. In our case report, we discuss the treatment of a 61-year-old male patient who had been complaining of abdominal pain for the past few years, was found to have a horseshoe kidney, a history of recurrent renal calculi with a non-functioning right side portion, and recurrent urinary tract infections (UTI) treated with open surgery. The patient symptomatically alleviated his symptoms at the three-month follow-up after open surgery; there were no complaints of discomfort or abdominal fullness, and the patient resumed daily routines.

## Introduction

The horseshoe kidney is the most common genitourinary fusion anomaly, occurring 1/400-1/800 times per 100,000 people [1]. Up to one-third of patients had malrotation, variable blood flow, and a tendency for ureteropelvic junction blockage [2]. Ureteropelvic junction blockage is considered to be caused by congenital strictures, a high ureteric insertion, an irregular ureteral course, crossed veins feeding the isthmus, or an aberrant ureteropelvic junction segment motility [3]. The most common complications of the horseshoe kidney are renal calculi and pelvic ureteric junction obstruction. The greater incidence of calculus formation was formerly assumed to be due to an elevated rate of infection, stasis, and blockages in these individuals. The majority of patients, according to the most current evaluations, have metabolic causes [4].

Because the collecting duct system as well as the ureters entering the bladder develop normally, a horseshoe kidney usually has no symptoms. Signs and symptoms of obstruction or infection may appear if the urinary flow is obstructed. Because the ascent of the fused kidneys is impeded by the origin of the inferior mesenteric artery from the abdominal aorta, the huge U-shaped kidney is frequently found in the hypogastrium, anterior to the lower lumbar vertebrae.

Here we are presenting the case of a 61-years-old male patient who presented with complaints of pain in the abdomen and was diagnosed with horseshoe kidney with multiple bilateral renal calculi with non-functioning right moiety. The patient in our case report had giant renal calculus with multiple large calculi noted in the bilateral kidneys with gross thinning of the cortices with recurrent urinary tract infections managed with open surgical stone removal as the removal was not amenable through extracorporeal shockwave lithotripsy (ESWL) or other treating modalities.

## Case presentation

A 61-year-old man presented with complaints of intermittent abdominal pain, burning and increased frequency of urination, lower abdominal pain, fullness in the bilateral flank region, and dysuria for the past four years. The patient was diagnosed as a case of right-sided non-functioning moiety with left-sided large renal calculi four years back. There is also a history of left-sided Double J (DJ) stenting a year before. The patient was assessed and looked into for increased frequency of urination and pain in the abdomen. Except for serum creatinine of 3.0 mg/dl and serum urea being 63 mg/dl, blood counts and liver function tests were normal. Urine routine microscopy revealed a large number of pus cells.

**Figure 1:**
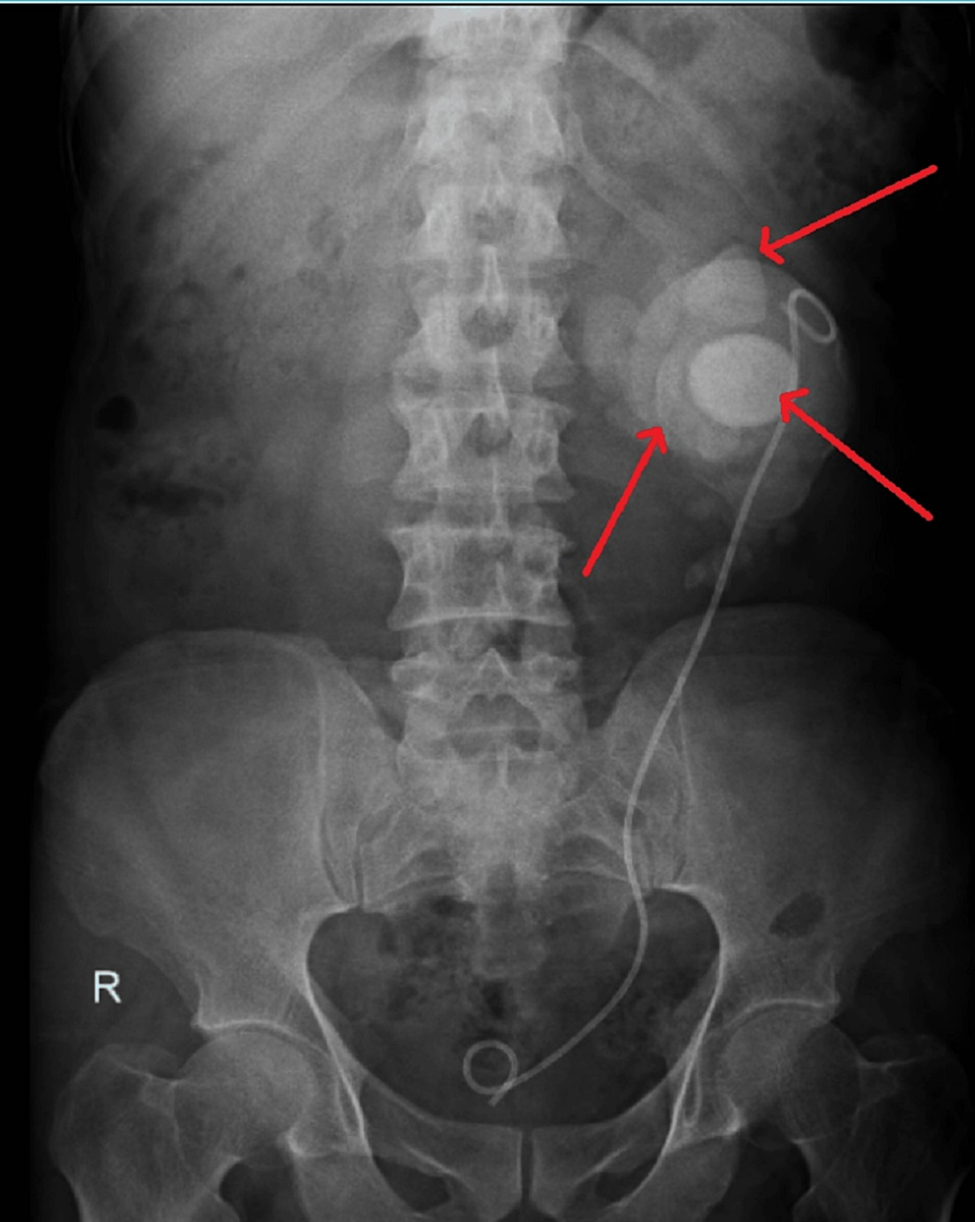
X-ray KUB The X-ray KUB (kidney, ureter, and bladder) is suggestive of multiple large left renal calculi with arrows pointing towards multiple calculi

**Figure 2:**
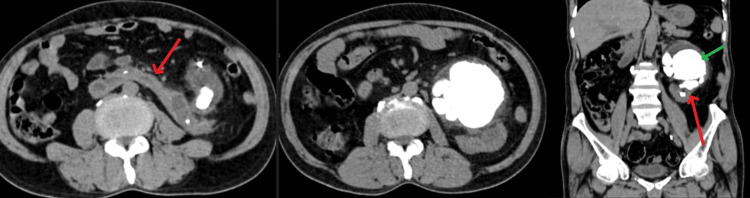
CT scan KUB CT scan KUB (kidney, ureter, and bladder) scan showing multiple large renal calculi with the largest calculi of 8 x 7.5 x 7.1 cm in a horseshoe kidney

Ultrasound abdomen revealed features suggestive of a horseshoe kidney with bilateral multiple renal calculi largest measuring 8 x 7 cm with right-sided moiety to be small and shrunken. X-ray KUB (kidney, ureter, and bladder) plain (Figure 1) was suggestive of multiple renal calculi in the left kidney. Hence, CT KUB plain (Figure 2) was done and it was suggestive of malrotation of both the kidneys and the horseshoe kidney. The right kidney measures 7 x 5.1 cm while the left kidney measures 12.3 x 7.8 cm. The isthmus measures 3.5 cm x 2.6 cm. Multiple large calculi were noted in the bilateral kidneys and isthmus largest of size 8 x 7.5 x 7.1 cm in the left kidney with gross thinning of the cortex in the upper and middle pole (3 mm) with maximum cortical thickness in the lower pole cortex measuring 15 mm. The right kidney also shows gross thinning of the cortex with a maximum cortical thickness of 4 mm in the lower pole. Minimal perinephric fat stranding noted. A DJ stent in situ is on the left side. 

Operative notes

**Figure 3:**
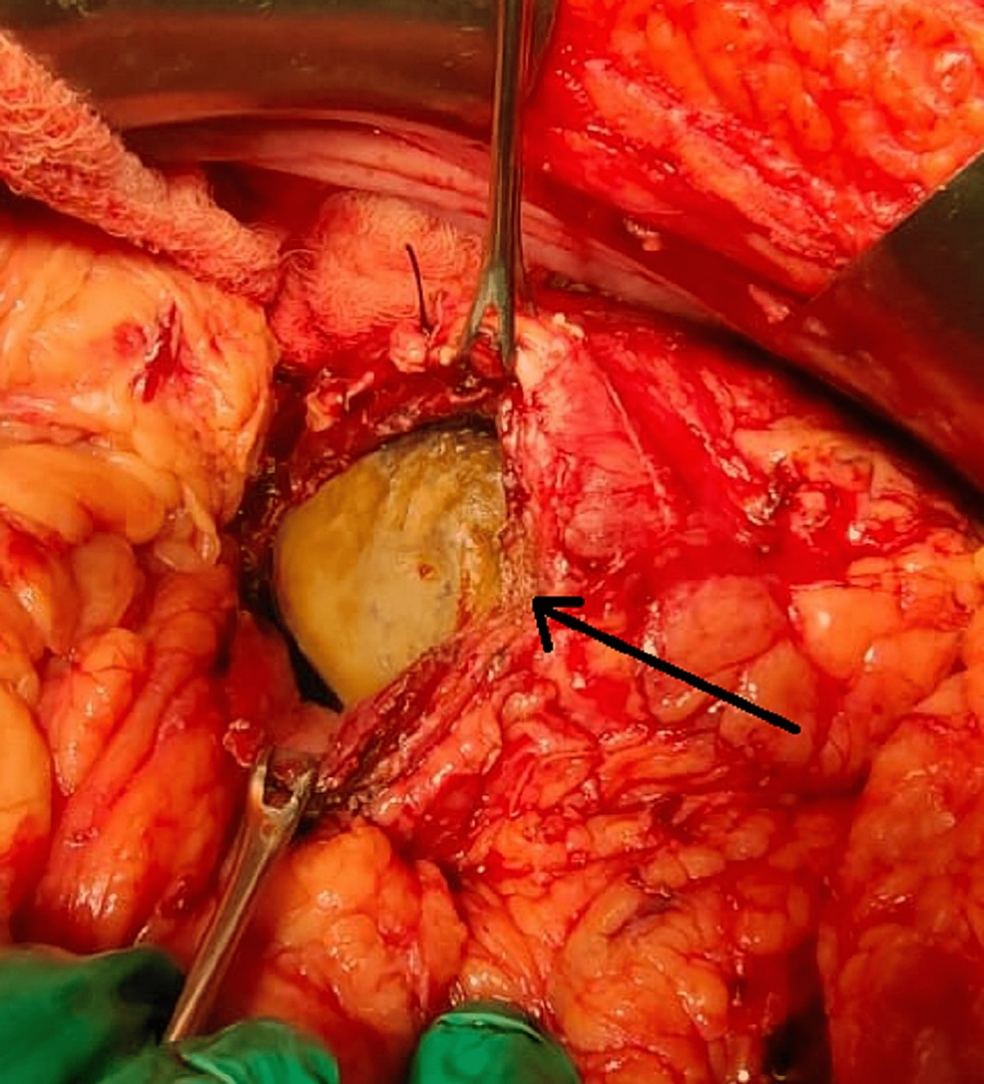
Intraoperative visualization of calculi after an incision over the upper pole of the kidney.

A lower midline incision was used to perform a left-sided open pyelolithotomy with DJ stenting. A low midline incision was taken, and the incision was deepened in layers to reach the left kidney, renal parenchyma was then separated from the surrounding tissues. Large calculi were noted and seen at the upper pole, Hence an oblique incision was made near the upper pole of approximately 3 cm (Figure 3).

**Figure 4:**
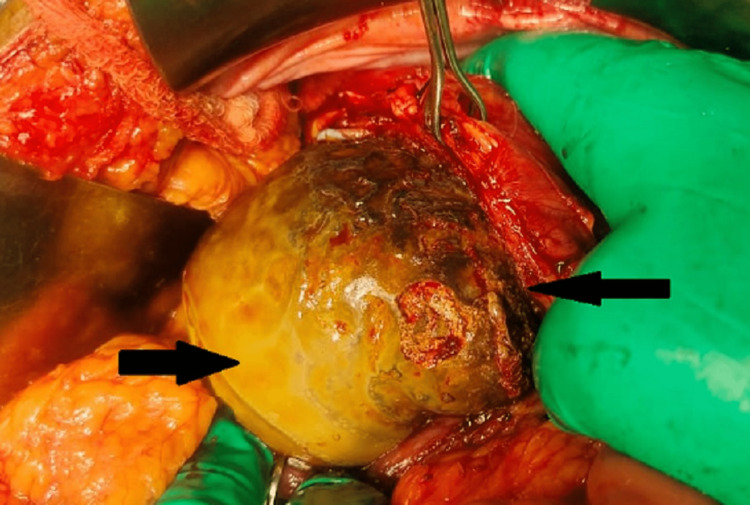
Intraoperative large calculi measuring approximately 8 cm visualized after oblique incision

Once the renal calculi were visualized, they were delivered using renal stone-holding forceps. The largest stone of approximately 8 x 7cm was delivered out (Figure 4) followed by 12 other smaller calculi.

**Figure 5:**
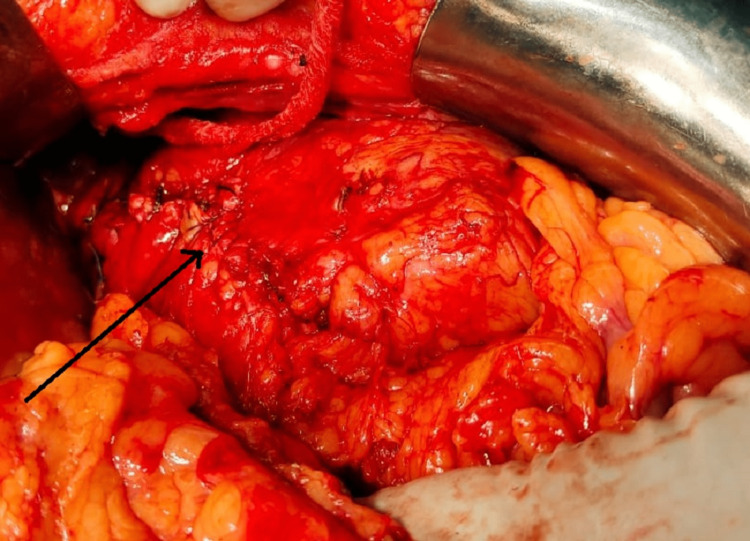
Closure of the renal pelvis after pyeloplasty.

**Figure 6:**
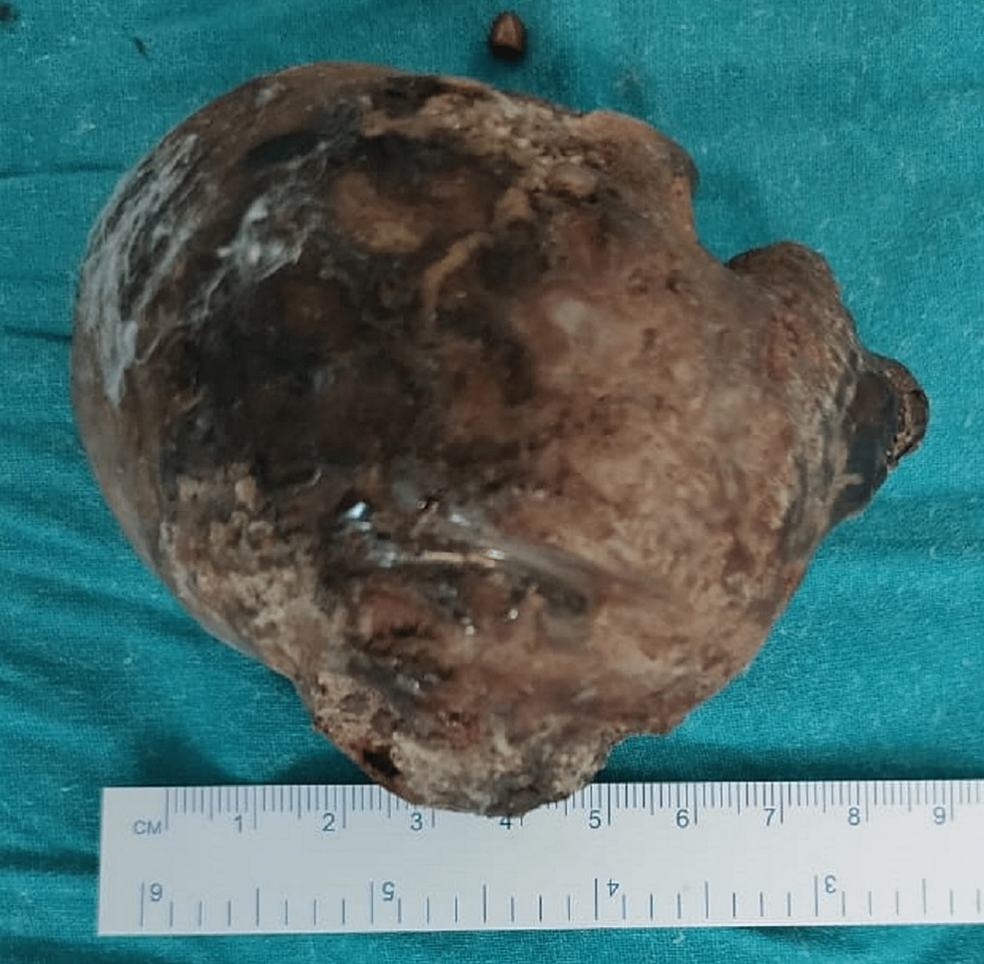
Largest renal calculi being 8 centimeters in dimension.

**Figure 7:**
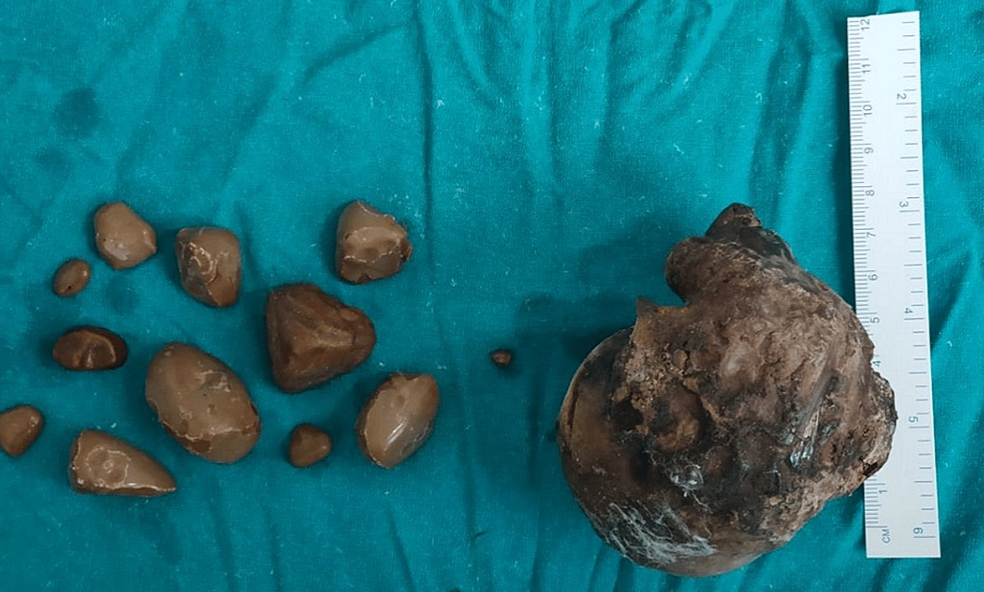
Multiple renal calculi with the largest calculi

Post stone extraction pyeloplasty was done and hemostasis was achieved (Figure 5) and large irregular stones were measured (Figures 6-7). The procedure was uneventful.

The patient was symptomatically eased throughout follow-up, with no complaints of abdominal discomfort or fullness.

## Discussion

The most frequent renal fusion anomaly, the horseshoe kidney, has a one in 400-600 incidence rate and a 2:1 male-female ratio [5]. The etiology of this disorder is characterized by abnormal renal ascent and malrotation. Horseshoe kidney is asymptomatic in at least one-third of individuals, and it is only seldom found by incidental imaging findings [6]. The inferior poles of the kidney fuse as it grows, and the inferior mesenteric artery stops it from ascending. An isthmus connects the kidneys, which might be a fibrous band or a thick rim of functioning renal tissue. Infection, calculi, blockage or tumor owing to an abnormal pelvic posture, and ureters are all clinical findings. A recent study by Domenech-Mateu et al. concluded that the cells from the posterior nephrogenic area of the epiblast that, due to an incomplete or abnormal migration across the primitive streak, remain in the midline and form the isthmus of the horseshoe kidney, a parenchymal structure, are those that migrate across the primitive streak in an abnormal or incomplete manner [7]. The renal isthmus, faulty lower pole arteries, and the kidney's unusual lower position are the main problems in pyeloplasty in this group. Horseshoe calculi and ectopic kidneys face particular obstacles in therapeutic decision-making and technical factors. In a few series, percutaneous nephrolithotomy is extremely effective, with an overall stone-free rate of 75-100 percent [8].

Raj et al. in their study of 37 patients identified with calculi in a horseshoe kidney concluded that due to the altered anatomical interactions of the fused renal units, percutaneous treatment of patients with renal calculi in a horseshoe kidney is technically difficult and frequently necessitates upper pole access and flexible nephroscopy [9]. Ergin et al. in their retrospective study concluded that the useful and efficient treatment options for urinary stones in kidneys with abnormal anatomy include percutaneous nephrolithotomy, laparoscopic pyelolithotomy, and retrograde intrarenal surgery (RIRS) [10]. 

In horseshoe kidneys with small and medium-sized calculi, RIRS could be employed since it is a safer and more effective minimally invasive method, in our study, there were multiple large calculi so these methods could not be employed. Hene, open pyelolithotomy was chosen as the treatment plan. The patient in this instance had a horse-shoe kidney with recurring numerous renal stones owing to bilateral pelvic-ureteric junction blockage and recurrent UTI. In a single situation, the patient was effectively handled with left-sided open pyelolithotomy considering open surgery a safe alternative in our resource-limited setting. Later on further visits, the patient was symptomatically eased, with no discomfort, and he had resumed his normal routine and job. The patient's follow-up kidney function tests were on the higher side of normal limits.

## Conclusions

In a horseshoe kidney patient with recurrent numerous renal calculi, a high index of suspicion for ureteropelvic junction obstruction should be applied. It requires a multi-modal strategy for treatment, which may include endoscopic and open surgical procedures as necessary. To reduce symptoms and prevent calculi from reappearing in these people, it is advised that the ureteropelvic junction blockage be treated. A follow-up protocol should be established when there is clinical evidence of a horseshoe kidney so that crucial time regarding the patient's overall renal function is not lost. We believe that open surgery still has a place in the treatment of renal calculi, even though it is currently rarely performed, due to the recent advances in the treatment of renal calculi, the majority of renal calculi can be managed with minimally invasive surgery. However, it is crucial to understand that a small group of patients with complex renal disease and those with anatomical anomalies will be better suitable for open surgeries.
